# Alleviative Effects of Ciprofol on Hepatic Ischemia/Reperfusion Injury Through Inhibiting Macrophage Polarization

**DOI:** 10.1002/iid3.70235

**Published:** 2025-07-29

**Authors:** Hanjian Chen, Heng Wen, Dongdong Tian, Huina Su, Ru Zhang, Lijia Zhang

**Affiliations:** ^1^ Department of Anesthesiology The First Affiliated Hospital of Zhejiang University School of Medicine Hangzhou China; ^2^ Department of Anesthesiology, School of Medicine Yan'an University Yan'an China; ^3^ Department of Surgical Anesthesiology The First Hospital of Yulin Yulin China

**Keywords:** ciprofol, hepatic ischemia/reperfusion injury, hypothesis, macrophage polarization, therapeutic

## Abstract

**Background:**

Previous studies have demonstrated the protective role of ciprofol against ischemia/reperfusion (I/R) injury, with the present investigation focusing on elucidating its effects on hepatic I/R injury.

**Methods:**

A hepatic I/R injury animal model was established, and macrophages were polarized using lipopolysaccharide (LPS) induction. Hepatic tissue damage and apoptosis were assessed through hematoxylin‐eosin and TUNEL staining. Liver function parameters, including aspartate aminotransferase (AST) and alanine aminotransferase (ALT), as well as pro‐inflammatory cytokine levels, were quantified using commercial assay kits. Macrophage polarization was evaluated via quantitative real‐time PCR, immunofluorescence, and immunoblotting, with flow cytometry additionally employed to assess cellular polarization. Pro‐inflammatory cytokine concentrations were also measured.

**Results:**

In the I/R model mice, ciprofol, comparable to the positive control propofol, and the macrophage eliminator gadolinium chloride (GdCl_3_) effectively attenuated inflammation and apoptosis, restored hepatic function, and inhibited macrophage polarization, as evidenced by reduced pro‐inflammatory cytokine levels. In LPS‐induced macrophages, ciprofol treatment decreased the proportion of CD86‐positive cells and the expression of macrophage polarization markers, alongside a reduction in pro‐inflammatory cytokine levels, mirroring the effects observed with propofol.

**Conclusion:**

These findings suggest that ciprofol exerts hepatoprotective effects against I/R injury by modulating macrophage polarization.

## Graphical abstract

This study investigates the protective effects of ciprofol on hepatic ischemia/reperfusion (I/R) injury, demonstrating that it reduces inflammation and apoptosis in liver tissue. Using a mouse model, ciprofol treatment was shown to suppress macrophage polarization and decrease pro‐inflammatory cytokine levels, similar to the effects observed with the positive control propofol. These findings suggest that ciprofol may offer a protective mechanism against liver I/R injury by inhibiting macrophage activation.

## 1 Introduction

Hepatic ischemia/reperfusion (I/R) injury, commonly encountered during hepatic surgery such as partial hepatectomy and liver transplantation, as well as in conditions associated with hypovolemic shock and hypoxia‐related diseases, significantly impairs liver function [1, 2]. As a critical factor contributing to early graft rejection, hepatic I/R injury is associated with elevated postoperative morbidity and mortality rates [3, 4]. Various therapeutic approaches have been developed to mitigate hepatic I/R injury, encompassing noninvasive pharmacological interventions and advanced techniques such as liver conditioning and machine perfusion [5]. The pathophysiological mechanisms underlying hepatic I/R injury primarily involve microcirculatory dysfunction, hypoxia, oxidative stress (OS), and cell death [6]. Additionally, increasing evidence has identified the involvement of extrahepatic monocyte‐derived macrophages in the pathogenesis of hepatic I/R injury, although the mechanisms regulating their response remain incompletely understood [7].

Macrophages, as the most abundant nonparenchymal cell type in the liver, are pivotal in innate immune responses and cellular functions linked to both inflammation and hepatic I/R injury [8]. Their phenotypic and functional profiles are dynamically influenced by the hepatic microenvironment, typically differentiating into two major subsets: classically activated (M1) macrophages, which are induced by lipopolysaccharide (LPS) and secrete pro‐inflammatory cytokines, and alternatively activated (M2) macrophages, which exhibit anti‐inflammatory and immunomodulatory properties [9]. These subsets exhibit contrasting functions during hepatic I/R injury, with the balance between M1 and M2 macrophages influencing disease outcomes [10, 11]. Therefore, identifying potential agents capable of modulating the M1/M2 macrophage ratio within the liver is essential for therapeutic intervention in hepatic I/R injury.

Natural‐derived compounds have garnered attention for their therapeutic potential in managing hepatic I/R injury due to their notable pharmacological efficacy [12]. Ciprofol (HSK3486), a 2,6‐disubstituted phenol derivative, exhibits high affinity for the gamma‐aminobutyric acid A (GABAA) receptor [13]. Previous randomized trials have established its efficacy in anesthesia induction and maintenance under various clinical conditions [14]. Furthermore, preliminary studies have suggested that ciprofol may confer neuroprotective effects against cerebral I/R injury [15]. Given its benefits in other I/R injury models, it is hypothesized that ciprofol may exert a protective role in hepatic I/R injury. Therefore, this study was conducted to investigate the hepatoprotective potential of ciprofol, presented in accordance with the ARRIVE Guidelines reporting checklist.

## 2 Materials and Methods

### 2.1 Ethics

The Animal Experimental Ethical Inspection Committee of the First Affiliated Hospital, Zhejiang University School of Medicine, granted ethical approval for this study (2023‐416). All animal procedures were strictly followed the guidelines of the China National Council on Laboratory Animal Welfare, with comprehensive measures taken to minimize animal distress.

### 2.2 Animal Model

The hepatic I/R injury model was established following a previously described protocol [11]. Male C57BL/6J mice (6–8 weeks old) were obtained from the Experimental Animal Center of the First Affiliated Hospital of Zhejiang University School of Medicine. The animals were housed under controlled temperature and humidity conditions with ad libitum access to sterile chow in a designated animal facility.

Mice were randomly assigned to one of the following experimental groups: Sham, IR, IR + propofol, IR + ciprofol, IR + Gadolinium chloride (GdCl_3_), and IR + ciprofol + GdCl_3_ (*n* = 3 per group). Drug administration involved intravenous infusion of propofol (40 mg/kg, BP1031, Merck Millipore, Germany), ciprofol (20 mg/kg, Haisco Pharmaceutical, China), or GdCl_3_ (10 mg/kg, G24750, Acmec Biochemical, China) before model induction [11, 16].

To establish the hepatic I/R injury model, mice were anesthetized and underwent midline laparotomy. The arterial and portal venous blood supply to the left and middle liver lobes was occluded using an atraumatic clip under an operating microscope, inducing partial hepatic warm ischemia for 1 h. Reperfusion was achieved by gently removing the clamp, followed by a 24‐h recovery period. Throughout the procedure, core body temperature was maintained at 32°C using a heating pad. Mice in the sham group underwent identical surgical procedures without hepatic ischemia. After reperfusion, mice were euthanized by cervical dislocation for subsequent analysis.

### 2.3 Hematoxylin‐Eosin (H&E) Staining

Liver tissue samples were fixed in 4% paraformaldehyde (P39200, Acmec Biochemical, China) and embedded in paraffin (P46563, Acmec Biochemical, China), followed by sectioning into 5 μm‐thick slices. After deparaffinization and rehydration, hematoxylin‐eosin (H&E) staining was performed using a commercially available kit (AC11917, Acmec Biochemical, China). The stained sections were then dehydrated, mounted, and examined under an optical microscope (Eclipse Ni‐U, Nikon, Japan) [17].

### 2.4 TUNEL Apoptosis Staining

Apoptosis in hepatic tissues was assessed using a TUNEL assay kit (C1086, Beyotime, China). Paraffin‐embedded sections were deparaffinized, followed by a 30‐min incubation with 100 μL proteinase K solution (P92300, Acmec Biochemical, China) at 37°C. Next, 100 μL DNase I reaction solution (D7073, Beyotime, China) was applied, followed by the addition of TdT enzyme and fluorescein‐labeling solutions (50 μL each) in the dark. Nuclei were subsequently stained with DAPI solution (C1005, Beyotime, China), and images were captured using a fluorescence microscope (Eclipse 50i, Nikon, Japan) [18].

### 2.5 Liver Function Test

Fresh blood samples were collected from each group of mice via the inferior vena cava and centrifuged at 3000 rpm for 10 min at 4°C to obtain serum. Serum levels of aspartate aminotransferase (AST, catalog no. C010‐1‐1) and alanine aminotransferase (ALT, catalog no. C009‐1‐1) were quantified using commercial assay kits from Jiancheng Bioengineering Institute (Nanjing, China), following the manufacturer's instructions.

### 2.6 Enzyme‐Labeled Immunosorbent Assay (ELISA)

The concentrations of pro‐inflammatory cytokine, including interleukin (IL)‐1β (catalog no. PI301), IL‐6 (catalog no. PI326), and tumor necrosis factor (TNF)‐α (catalog no. PT512), were measured using corresponding enzyme‐linked immunosorbent assay (ELISA) kits from Beyotime, strictly adhering to the manufacturer's protocols. Serum samples were incubated with biotinylated antibody solution, horseradish peroxidase (HRP)‐conjugated streptavidin, and the visualization substrate. The reaction was terminated with a stop solution, and the optical density (OD) at 450 nm (A_450_) was recorded using an iMark microplate reader (E0225, Beyotime, China) [19].

### 2.7 Cell Culture and Intervention

The mouse macrophage cell line RAW264.7 (C7505, Beyotime, China) was cultured in high‐glucose Dulbecco's modified Eagle's medium (DMEM, D0822, Merck Millipore, Germany) supplemented with 10% fetal bovine serum (FBS, 12103 C, Merck Millipore, Germany) under standard conditions (37°C, 5% CO_2_).

To induce M1 macrophage polarization, RAW264.7 cells were treated with lipopolysaccharide (LPS, 100 ng/mL, ST1470, Beyotime, China) for 24 h. Subsequently, cells were exposed to either propofol (50 μM) or ciprofol (20 μg/mL) for an additional 24 h.

### 2.8 Immunofluorescence Assay

Paraffin‐embedded liver tissue sections were rehydrated and deparaffinized, followed by antigen retrieval using 0.01 M Tris‐EDTA solution (P0084, Beyotime, China). For immunostaining, induced macrophages were fixed in 5% goat serum (C0265, Beyotime, China). Primary antibodies targeting NOS2 and CSF2 (Table 1) were incubated with the tissue sections or cells overnight at 4°C. The samples were then treated with the appropriate secondary antibodies at room temperature for 1 h. DAPI staining solution was subsequently applied to label cell nuclei, and an anti‐fade reagent (P0126, Beyotime, China) was used before coverslip sealing. The stained sections were observed using a fluorescence microscope (Eclipse 50i, Nikon, Japan), and images were captured.

**Table 1:** Information of antibodies.

Identifier	Catalog no.	Conjugate	Application	Dilution	Note
Rabbit iNOS Antibody (Alexa Fluor 488)	NB300‐605AF488	Alexa Fluor 488	Immunofluorescence	1:100	Green fluorescence
Rabbit Alexa Fluor 647 Anti‐iNOS antibody (EPR16635)	ab209027	Alexa Fluor 647	Immunofluorescence	1:100	Red fluorescence
Mouse GM‐CSF Antibody (83308) (Alexa Fluor 488)	IC5181G‐100UG	Alexa Fluor 488	Immunofluorescence	1:100	Green fluorescence
CD86 Recombinant Rabbit Monoclonal Antibody (313), PE	MA5‐52361	phycoerythrin	Flow cytometry	1:100	Green fluorescence
Anti‐iNOS antibody (EPR16635)	ab178945	—	Immunoblotting	1:1000	—
Anti‐GM‐CSF antibody	ab9741	—	Immunoblotting	1:1000	—
Anti‐GAPDH antibody (EPR16891)—loading control	ab181602	—	Immunoblotting	1:10000	—
Goat anti‐rabbit IgG H&L	ab6702	—	Immunofluorescence	1:10000	—
Goat anti‐mouse IgG H&L	ab6708	—	Immunofluorescence	1:10000	—
Goat anti‐rabbit IgG H&L	ab205718	HRP	Immunoblotting	1:2000	—

### 2.9 Flow Cytometry

Induced macrophages were seeded into a 6‐well plate at a density of 5 × 10^5^ cells per well and cultured for 4 days. After incubation, the cells were trypsinized, scraped from the plate, and centrifuged. The cell pellet was then resuspended in 1% bovine serum albumin (BSA, ST023, Beyotime, China) and incubated at room temperature for 30 min. Subsequently, the macrophages were stained with anti‐CD86 antibody (Table 1) at ambient temperature for 30 min. Following two washes with phosphate‐buffered saline (PBS), the cells were resuspended in 1% BSA and analyzed using a Guava flow cytometer (Cytek Biosciences, Fremont, CA, USA) and processed with Guava Soft 3.1.1 (Cytek Biosciences, USA) [20].

### 2.10 Quantitative Real‐Time PCR

Total RNA was extracted from liver tissue and macrophages using TriZol reagent (15596‐026, Invitrogen, Carlsbad, CA, USA), and RNA concentrations were determined with a spectrophotometer (ND‐2000, ThermoFisher, Waltham, MA, USA). Reverse transcription was performed using a cDNA synthesis kit (K1622, ThermoFisher, USA) to generate complementary DNA (cDNA), which was then utilized for quantitative PCR (qPCR) analysis with qPCR SYBR Green Mix (11201ES03, Yeason, Shanghai, China). Relative mRNA expression levels were calculated using the 2^−^^ΔΔCT^ method, with GAPDH serving as the housekeeping control [21, 22]. Primer sequences used in the qPCR assay are listed in Table 2.

**Table 2:** Primers for the qPCR assay.

Target gene	Forward primer (5′‐3′)	Reverse primer (5′‐3′)
*Nos2*	AGGACATTAACAACAACGTG	GACTCTTAGGGTCATCTTGTA
*Csf2*	GCTGCAGAATTTACTTTTCC	GGTGGTAACTTGTGTTTCA
*Il1b*	CTGAACTCAACTGTGAAATG	AAGTCAATTATGTCCTGACC
*Gapdh*	GCTTAGGTTCATCAGGTAAA	TGACAATCTTGAGTGAGTTG

### 2.11 Immunoblotting

Protein samples were extracted from liver tissues and macrophages using radio‐immunoprecipitation assay (RIPA) buffer (P0013B, Beyotime, China) supplemented with protease and phosphatase inhibitors (P1045, Beyotime, China) at 4°C for 30 min. Protein concentrations were quantified using a bicinchoninic acid (BCA) assay kit (P0012S, Beyotime, China) according to the manufacturer's protocol.

Equal amounts of protein (30 μg) were separated by SDS‐PAGE and subsequently transferred onto polyvinylidene difluoride (PVDF) membranes. The membranes were blocked with 5% skimmed milk and incubated with primary antibodies (Table 1) at 4°C overnight. Following incubation, membranes were exposed to HRP‐conjugated secondary antibodies at room temperature for 1 h. Protein bands were visualized using an enhanced chemiluminescence (ECL) kit (P0018S, Beyotime, China) and captured using the ChemiDoc imaging system (Bio‐Rad, Hercules, CA, USA). The resulting images were analyzed with ImageJ 1.42 software (National Institutes of Health, Bethesda, MD, USA) [23].

### 2.12 Statistical Analyses

Statistical analyses were performed using GraphPad Prism 8.0.2 (GraphPad Inc., La Jolla, CA, USA). Data from three independent experiments were presented as mean ± standard deviation. Differences between groups were evaluated using one‐way analysis of variance (ANOVA) followed by a post‐hoc test. A *p* value of less than 0.05 was considered statistically significant.

## 3 Results

### 3.1 Effects of Ciprofol on the Hepatic I/R Injury‐Induced Damage and Apoptosis in the Liver

**Figure 1:**
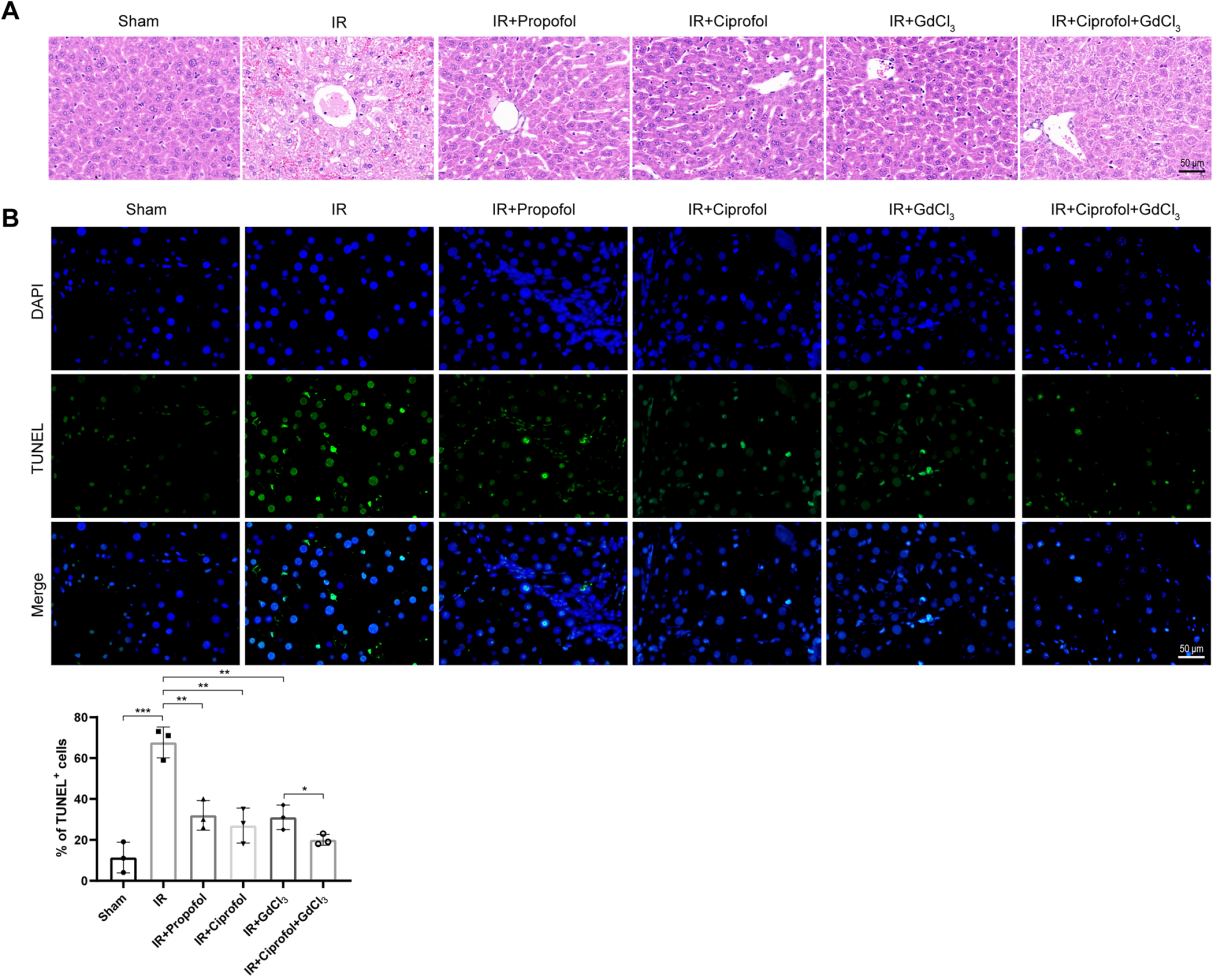
Effects of ciprofol on hepatic ischemia/reperfusion injury‐induced damage and apoptosis. (A) Representative H&E staining results showing liver tissue morphology following the indicated treatments. (B) Representative TUNEL staining results depicting hepatic apoptosis and quantification of TUNEL‐positive cells in each group (*F* = 23.87, *p* < 0.0001). (*n* = 3 per group). Data from three independent experiments are expressed as mean ± standard deviation. Statistical significance between linked groups is indicated by asterisks (*p < 0.05, **p < 0.01, ***p < 0.001).

The hepatic I/R injury model was employed to investigate the potential hepatoprotective effects of ciprofol. The outcomes of hematoxylin‐eosin (H&E) and TUNEL staining are presented in Figure 1. H&E staining revealed intact hepatic architecture in the Sham group, while the IR model group exhibited significant tissue damage and inflammatory cell infiltration (Figure 1A). Notably, treatment with propofol, ciprofol, or GdCl_3_ markedly mitigated hepatic damage and inflammation. Among these interventions, the combined treatment of ciprofol and GdCl_3_ demonstrated a superior alleviative effect (Figure 1A). TUNEL staining further confirmed that administration of propofol, ciprofol, or GdCl_3_ significantly reduced I/R‐induced hepatocyte apoptosis, as evidenced by the decreased percentage of TUNEL‐positive cells (Figure 1B, *p* < 0.01). The combination of ciprofol and GdCl_3_ exhibited a more pronounced antiapoptotic effect compared to individual treatments (Figure 1B, *p* < 0.05). These results indicate that ciprofol effectively attenuates hepatic I/R‐induced tissue damage and apoptosis, particularly when combined with GdCl_3_.

### 3.2 Effects of Ciprofol on the Liver Function‐Related Parameters and Inflammatory Cytokines in Hepatic I/R Injury‐Modeled Mice

**Figure 2:**
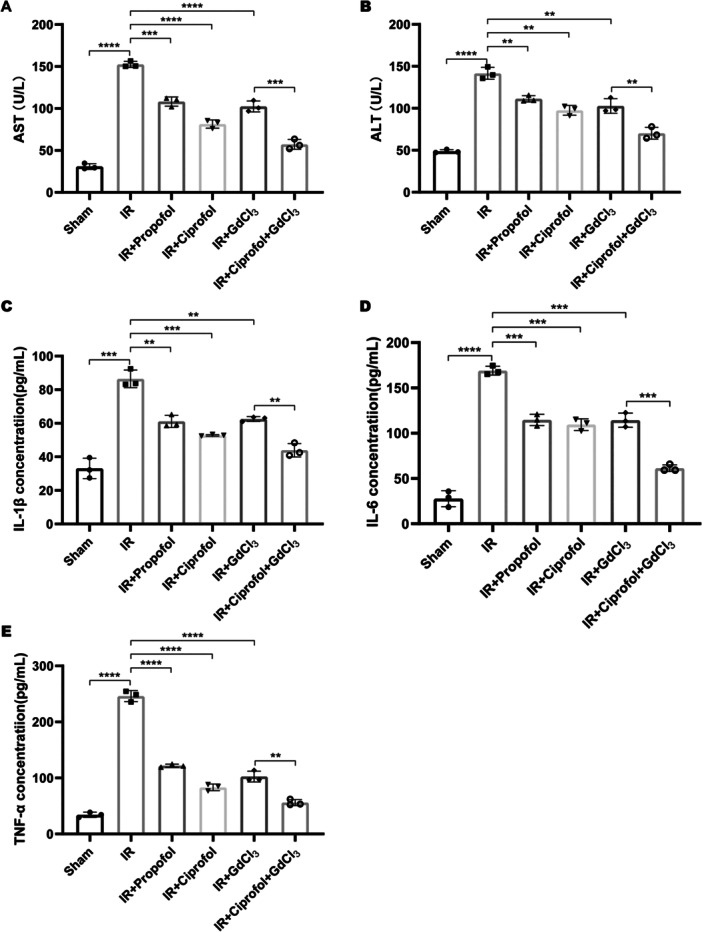
Effects of ciprofol on liver function parameters and inflammatory cytokines in I/R‐injured mice. (A and B) AST (A) and ALT (B) levels in I/R model mice (A: *F* = 210.9, *p* < 0.0001; B: *F* = 83.19, *p* < 0.0001). (C–E) Serum concentrations of IL‐1β (C), IL‐6 (D), and TNF‐α (E) in I/R model mice (C: *F* = 63.09, *p* < 0.0001; D: *F* = 168.5, *p* < 0.0001; E: *F* = 361.7, *p* < 0.0001). (*n* = 3 per group). Data from three independent experiments are presented as mean ± standard deviation. Statistical significance between groups is denoted as **p < 0.01, ***p < 0.001, ****p < 0.0001.

To assess the efficacy of the hepatic I/R injury model, the concentrations of liver function parameters AST and ALT were measured. The results indicated that I/R induction significantly increased the levels of both AST and ALT (Figure 2A,B, *p* < 0.0001), confirming the successful establishment of the injury model. Treatment with propofol, ciprofol, or GdCl_3_ independently led to a marked reduction in the levels of these enzymes (Figure 2A,B, *p* < 0.01), with the most pronounced effect observed following the combined administration of ciprofol and GdCl_3_ (Figure 2A,B, *p *< 0.01). To evaluate the inflammatory response, serum levels of pro‐inflammatory cytokines IL‐1β, IL‐6, and TNF‐α were quantified using ELISA. I/R injury significantly elevated the concentrations of all three cytokines (Figure 2C–E, *p* < 0.001). Treatment with propofol, ciprofol, or GdCl_3_ effectively suppressed the increase in cytokine levels (Figure 2C–E, *p* < 0.01). Notably, the combination of ciprofol and GdCl_3_ yielded a more substantial decrease in cytokine levels compared to monotherapy (Figure 2C–E, *p* < 0.01). These results indicate that ciprofol has a significant capacity to restore liver function and alleviate inflammatory responses in the I/R injury model.

### 3.3 Effects of Ciprofol on the Macrophage Polarization in the Liver Tissue of Modeled Mice

**Figure 3:**
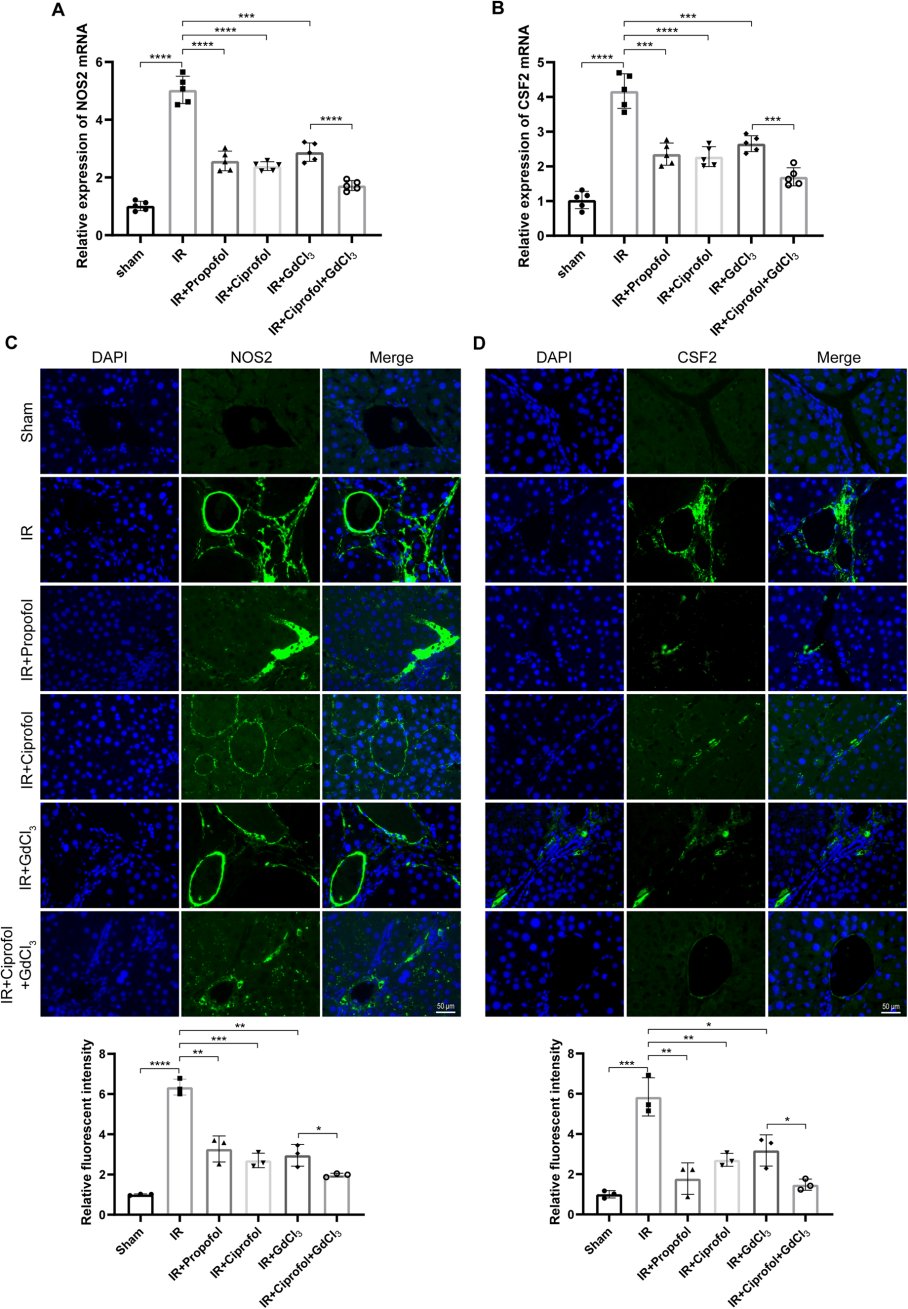
Effects of Ciprofol on macrophage polarization markers in liver tissue of I/R model mice. (A and B) Relative mRNA expression levels of *NOS2* (M1 marker; A) and *CSF2* (driver of M1 polarization; B) in liver tissue following indicated treatments (A: *F* = 108.0, *p* < 0.0001; B: *F* = 54.47, *p* < 0.0001). (C and D) Mean fluorescence intensity of NOS2 (C) and CSF2 (D) in liver tissue with indicated interventions (C: *F* = 58.67, *p* < 0.0001; D: *F* = 23.69, *p* < 0.0001). (*n* = 3 per group). Data are expressed as mean ± standard deviation from three independent experiments. Statistical significance between groups is indicated by asterisks (*p < 0.05, **p < 0.01, ***p < 0.001, ****p < 0.0001).

**Figure 4:**
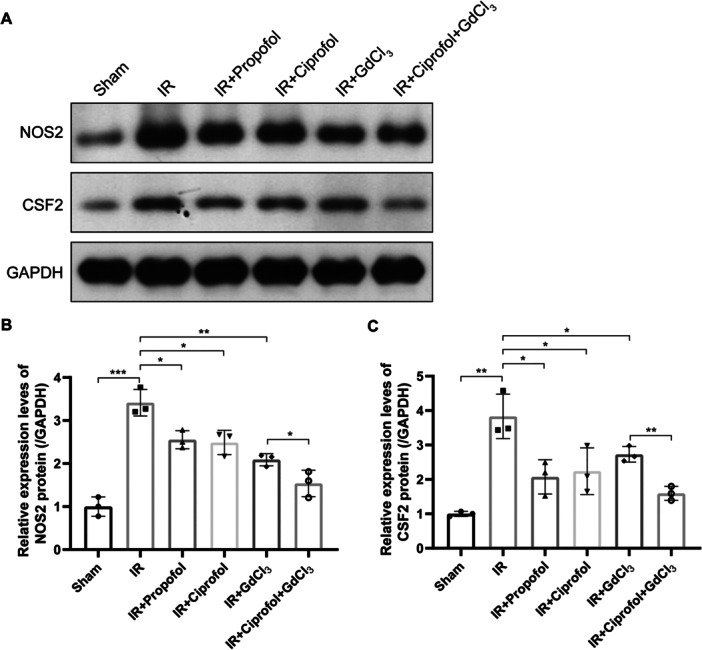
Effects of ciprofol on the protein expression of macrophage polarization markers in liver tissue of I/R model mice. (A–C) Protein expression levels of NOS2 (M1 marker; B) and CSF2 (driver of M1 polarization; C) following indicated treatments (B: *F* = 33.59, *p* < 0.0001; C: *F *= 13.98, *p* < 0.0001). (*n* = 3 per group). Data from three independent tests are presented as mean ± standard deviation, with statistical significance denoted as (*p < 0.05, **p < 0.01, ***p < 0.001).

To investigate macrophage polarization in the hepatic tissue of I/R model mice, the mRNA expression levels of *Nos2* (an M1 macrophage marker) and *Csf2* (a driver of M1 polarization) were measured. qPCR analysis revealed that I/R modeling significantly increased the expression levels of both *Nos2* and *Csf2*, while treatment with propofol, ciprofol, or GdCl_3_ significantly reduced these mRNA levels (Figure 3A,B, *p* < 0.001). Furthermore, the combined administration of ciprofol and GdCl_3_ demonstrated a more pronounced reduction in the mRNA levels of these markers (Figure 3A,B, *p* < 0.001). Immunofluorescence staining further corroborated these findings. The mean fluorescence intensity of both Nos2 and Csf2 in hepatic tissue significantly increased following I/R modeling, whereas interventions with propofol, ciprofol, or GdCl_3_ substantially diminished the fluorescence intensity (Figure 3C,D, *p* < 0.05). The most evident reduction was observed when ciprofol and GdCl_3_ were administered in combination. Consistently, immunoblotting results aligned with the aforementioned observations. The protein expression levels of Nos2 and Csf2 were significantly elevated in the I/R model, while treatment with propofol, ciprofol, or GdCl_3_ markedly decreased their protein levels (Figure 4A–C, *p* < 0.05). Again, the combined treatment of ciprofol and GdCl_3_ demonstrated superior efficacy in reducing the protein expression of these polarization markers. These results indicate that ciprofol may exert its protective effects against hepatic I/R injury by modulating macrophage polarization.

### 3.4 Effects of Ciprofol on the Expression of Macrophage Polarization Markers and Pro‐Inflammatory Cytokines in LPS‐Induced Macrophages

**Figure 5:**
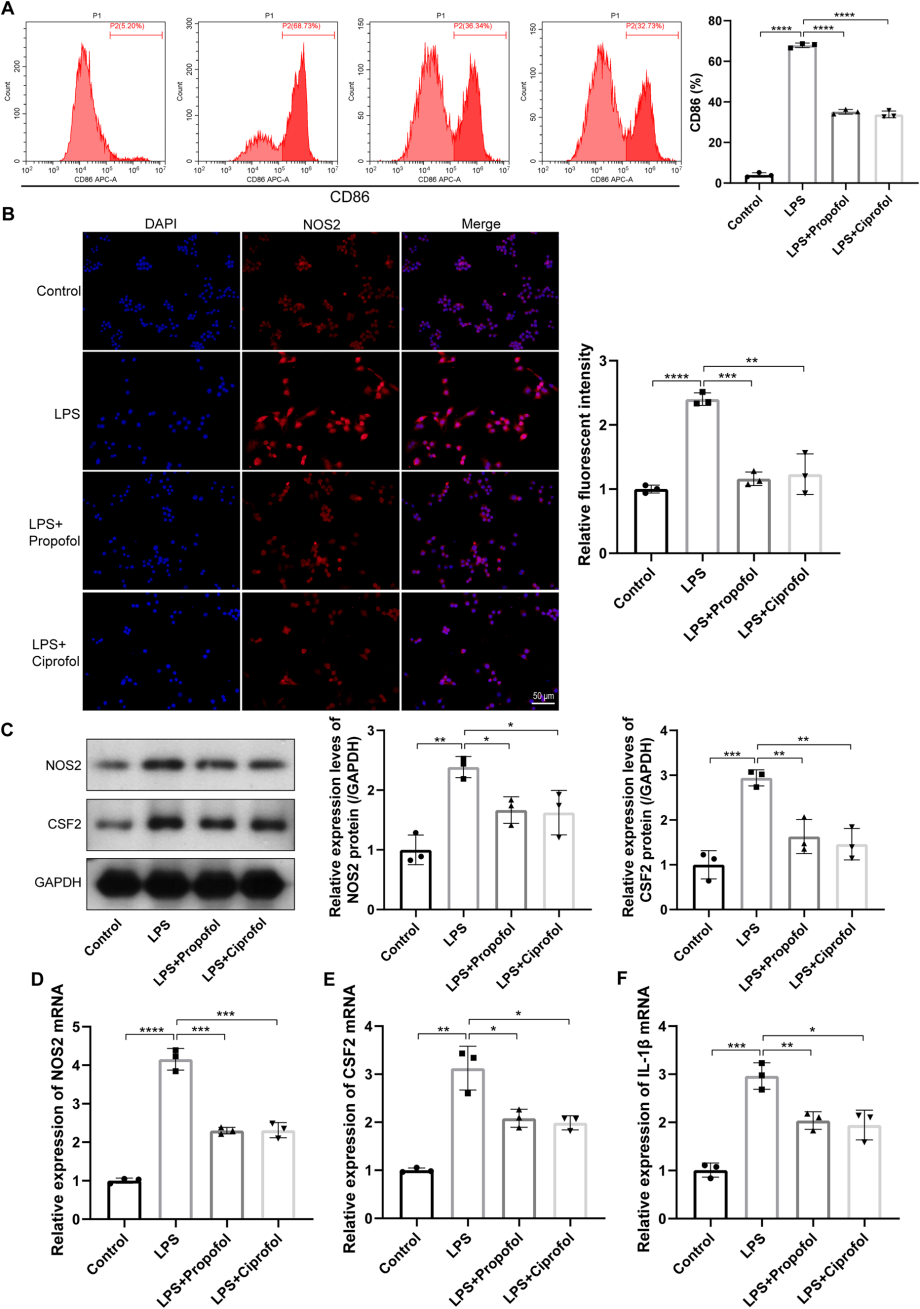
Effects of ciprofol on macrophage polarization markers and pro‐inflammatory cytokines in macrophages. (A) Percentage of CD86‐positive cells in macrophages after indicated interventions (*F* = 1230, *p* < 0.0001). (B) Relative fluorescence intensity of NOS2 in macrophages with indicated treatments (*F* = 39.91, *p* < 0.0001). (C) Relative protein expression levels of NOS2 (*F* = 13.69, *p* < 0.0001) and CSF2 (*F *= 20.81, *p* < 0.0001) in macrophages after interventions. (D and E) Relative mRNA expression levels of *NOS2* (M1 marker; D) and *CSF2* (driver of M1 polarization; E) in macrophages (D: *F* = 155.7, *p* < 0.0001; E: *F* = 33.89, *p* < 0.0001). (F) Relative mRNA levels of *IL‐1β* in macrophages with indicated treatments (*F* = 33.85, *p* < 0.0001). Data from three independent experiments are presented as mean ± standard deviation, with statistical significance denoted as (*p < 0.05, **p < 0.01, ***p < 0.001, ****p < 0.0001).

A cellular model of LPS‐induced macrophages was developed to investigate the modulatory effects of ciprofol on macrophage polarization. The percentage of CD86‐positive cells, representing M1 macrophage polarization, was quantified following the interventions. LPS stimulation significantly increased the proportion of CD86‐positive cells, whereas treatment with propofol or ciprofol markedly reduced this percentage (Figure 5A, *p *< 0.0001). Immunofluorescence analysis further demonstrated that both propofol and ciprofol significantly attenuated the LPS‐induced increase in the mean fluorescence intensity of CD86 in macrophages (Figure 5B, *p *< 0.01). Quantitative analysis of macrophage polarization markers revealed that LPS exposure significantly elevated the protein and mRNA expression levels of *NOS2* and *CSF2*. In contrast, propofol and ciprofol treatments effectively reduced the expression of these markers in macrophages following LPS induction (Figure 5C–E, *p* < 0.05). Additionally, *Il1b* levels in macrophages were quantified, indicating that LPS stimulation markedly increased Il1b expression, a trend significantly reversed by propofol and ciprofol (Figure 5F, *p* < 0.05).

**Figure 6:**
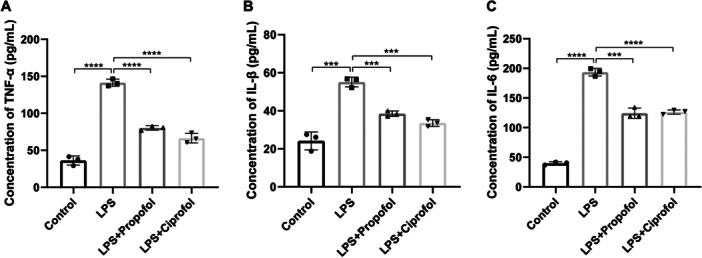
Effects of ciprofol on inflammatory cytokine levels in macrophages. (A–C) Concentration of IL‐1β (A), IL‐6 (B), and TNF‐α (C) in LPS‐induced macrophages following indicated treatments (A: *F* = 209.2, *p* < 0.0001; B: *F* = 60.43, *p* < 0.0001; C: *F* = 350.4, *p* < 0.0001). Data from three independent tests are expressed as mean ± standard deviation, with statistical significance indicated as (***p < 0.001, ****p < 0.0001).

Furthermore, the concentrations of inflammatory cytokines (TNF‐α, IL‐1β, and IL‐6) were assessed in macrophages following LPS induction and subsequent treatment with propofol or ciprofol. The results indicated that LPS significantly elevated cytokine levels, while both propofol and ciprofol effectively counteracted these inflammatory responses (Figure 6A–C, *p* < 0.001).

These results suggest that ciprofol modulates macrophage polarization by inhibiting M1 polarization, thereby reducing pro‐inflammatory cytokine production in LPS‐induced macrophages.

## 4 Discussion

I/R injury represents a surgical emergency characterized by high morbidity and mortality, primarily driven by ischemia‐induced organ damage, subsequently exacerbated by reperfusion‐related secondary tissue injury [24]. Extensive research has consistently demonstrated that this pathology involves the release of pro‐inflammatory cytokines and the production of inflammatory mediators [25]. Notably, ciprofol has been reported to ameliorate myocardial I/R injury by mitigating OS and inflammation [26], which aligns with the present findings indicating that ciprofol effectively suppresses the production of pro‐inflammatory cytokines associated with hepatic I/R injury.

Hepatic macrophage polarization has been implicated as a key factor in the pathogenesis of hepatic I/R injury [27]. This evidence prompted an investigation into whether ciprofol exerts its protective effects against hepatic I/R injury through the attenuation of inflammation and regulation of macrophage polarization. The findings of this study demonstrate that ciprofol, comparable to the positive control propofol, significantly restores liver function, reduces inflammation and apoptosis in vivo, and inhibits macrophage polarization in vitro. These results underscore the pivotal role of ciprofol in mitigating hepatic I/R injury and suggest that the modulation of macrophage polarization constitutes a potential mechanism underlying its hepatoprotective effects.

Ciprofol, an innovative compound independently developed in China, is primarily utilized for inducing sedation and anesthesia during non‐tracheal intubation procedures, the induction and maintenance of general anesthesia, and sedation in intensive care settings [28]. Beyond its anesthetic applications, ciprofol has demonstrated efficacy in various physiological contexts. Notably, it has been reported to mitigate electroconvulsive shock‐induced cognitive deficits in depressive rats by modulating aerobic glycolysis [29] and to alleviate remifentanil‐induced hyperalgesia in both the spinal cord and hippocampus [29]. In the context of I/R injury, ciprofol has been shown to protect the brain from I/R‐induced damage through Nrf2‐mediated anti‐OS mechanisms [30, 31] and to reduce isoproterenol‐induced OS, inflammatory response, and cardiomyocyte apoptosis [26]. Despite these findings, the potential protective effects of ciprofol against hepatic I/R injury remain inadequately explored. This study aimed to address this gap by evaluating the efficacy of ciprofol in hepatic I/R injury. The results demonstrated that ciprofol significantly reduced liver function‐related parameters (ALT and AST) and decreased the levels of pro‐inflammatory cytokines, indicating its antioxidative stress and anti‐inflammatory properties. These outcomes are consistent with previous evidence suggesting that ciprofol mitigates myocardial I/R injury by reducing oxidative stress, inflammation, and apoptosis via activation of the Sirt1/Nrf2 pathway [26].

The pathogenesis of hepatic I/R injury is complex, involving multiple regulatory factors, including OS and cytokine production [32]. OS is defined as an imbalance between reactive oxygen species (ROS) and the antioxidant defense system, characterized by elevated pro‐inflammatory cytokine production [33, 34, 35]. IL‐1β, a pro‐inflammatory cytokine released from I/R‐injured liver tissue, plays a pivotal role in ROS production and inflammatory responses; its deficiency has been associated with reduced ROS levels and mitigated inflammation [36]. IL‐6, a multifunctional cytokine with a four‐helix‐bundle structure, significantly contributes to the acute phase response and infection defense mechanisms within the liver [37, 38]. TNF‐α, a critical pro‐inflammatory mediator primarily produced by hepatic Kupffer cells, initiates the hepatic inflammatory cascade by promoting neutrophil recruitment following I/R‐induced liver injury [39]. To investigate the impact of ciprofol on hepatic I/R injury, liver function parameters and pro‐inflammatory cytokine levels were systematically evaluated. The findings revealed that ciprofol effectively suppressed ALT and AST concentrations and reduced the production of IL‐1β, IL‐6, and TNF‐α. These results indicate that ciprofol exerts antioxidative and anti‐inflammatory effects in the context of hepatic I/R injury.

Macrophages, as the predominant cellular components of the liver, are generally classified into two phenotypes: M1, which exhibits a pro‐inflammatory role during liver injury, and M2, which plays an anti‐inflammatory role in liver repair [40, 41]. Emerging evidence indicates that macrophage polarization exerts a significant influence on the initiation and progression of liver diseases, including hepatic I/R injury [42]. For example, Liraglutide has been shown to modulate macrophage polarization, thereby alleviating hepatic I/R injury [11]. Similarly, PGC‐1α has been reported to inhibit M2 macrophage polarization following hepatic I/R injury [10], while polydatin has demonstrated protective effects against hepatic I/R injury by remodeling macrophage polarization [43]. In the present study, the effects of ciprofol on macrophage polarization were specifically examined. The findings revealed that ciprofol attenuates I/R‐induced M1 macrophage polarization and significantly reduces the expression of the M1‐specific marker NOS2 both in I/R‐modeled mice in vitro and in LPS‐induced macrophages in vivo.

These results suggest that ciprofol may confer hepatoprotective effects by inhibiting M1 polarization. This observation aligns with previous studies indicating that ciprofol modulates immune responses favorably during injury. Notably, ciprofol has been reported to protect cardiac tissue by upregulating HIF‐1α expression, thereby preventing ferroptosis in cardiomyocytes during ischemia‐reperfusion injury [31].

Despite the positive outcomes demonstrated in this study, several limitations warrant consideration. Firstly, the research predominantly utilized animal and cell models, which may limit the translational applicability of the findings to clinical practice. Future investigations should aim to validate the efficacy and safety of ciprofol within a clinical context. Secondly, although the beneficial effects of ciprofol on liver function, inflammation, and macrophage polarization were observed, the precise molecular pathways through which ciprofol exerts its actions remain to be elucidated. Identifying these pathways will be an important focus of subsequent research. Furthermore, the relatively small sample size may constrain the generalizability of the results, highlighting the need for studies with larger sample sizes to enhance external validity. Additionally, exploring the effects of varying dosages and administration modes of ciprofol, along with more comprehensive mechanistic studies, would provide deeper insights into its therapeutic potential.

## 5 Conclusion

In conclusion, ciprofol protects the liver against I/R‐induced inflammation, apoptosis, and macrophage polarization in both mice and LPS‐induced macrophages. Nevertheless, the specific downstream signaling pathways mediating these protective effects remain unclear, necessitating further investigation. Additionally, research focusing on the time course and optimal concentrations of ciprofol for mitigating I/R injury is essential to optimize its clinical application.

## Author Contributions

**Hanjian Chen:** conceptualization, formal analysis, and writing – original draft. **Heng Wen:** conceptualization, data curation, and writing – original draft. **Dongdong Tian:** Investigation and methodology. **Huina Su:** methodology, project administration, and resources. **Ru Zhang:** visualization. **Lijia Zhang:** conceptualization, writing – review and editing.

## Ethics Statement

The animal study protocol was approved by the Animal Experimental Ethical Inspection of the First Affiliated Hospital, Zhejiang University School of Medicine (2023‐416).

## Conflicts of Interest

The authors declare no conflicts of interest.

## Data Availability

The data sets used and/or analyzed in the current study are available from the corresponding author upon reasonable request.
